# Estimation of causal effects of a time-varying exposure at multiple time points through multivariable mendelian randomization

**DOI:** 10.1371/journal.pgen.1010290

**Published:** 2022-07-18

**Authors:** Eleanor Sanderson, Tom G. Richardson, Tim T. Morris, Kate Tilling, George Davey Smith

**Affiliations:** 1 MRC Integrative Epidemiology Unit at the University of Bristol, Bristol, United Kingdom; 2 Population Health Sciences, Bristol Medical School, University of Bristol, Bristol, United Kingdom; 3 Novo Nordisk Research Centre, Headington, Oxford, United Kingdom; University Hospital of the Canton Vaud (CHUV), SWITZERLAND

## Abstract

Mendelian Randomisation (MR) is a powerful tool in epidemiology that can be used to estimate the causal effect of an exposure on an outcome in the presence of unobserved confounding, by utilising genetic variants as instrumental variables (IVs) for the exposure. The effect estimates obtained from MR studies are often interpreted as the lifetime effect of the exposure in question. However, the causal effects of some exposures are thought to vary throughout an individual’s lifetime with periods during which an exposure has a greater effect on a particular outcome. Multivariable MR (MVMR) is an extension of MR that allows for multiple, potentially highly related, exposures to be included in an MR estimation. MVMR estimates the direct effect of each exposure on the outcome conditional on all the other exposures included in the estimation. We explore the use of MVMR to estimate the direct effect of a single exposure at different time points in an individual’s lifetime on an outcome. We use simulations to illustrate the interpretation of the results from such analyses and the key assumptions required. We show that causal effects at different time periods can be estimated through MVMR when the association between the genetic variants used as instruments and the exposure measured at those time periods varies. However, this estimation will not necessarily identify exact time periods over which an exposure has the most effect on the outcome. Prior knowledge regarding the biological basis of exposure trajectories can help interpretation. We illustrate the method through estimation of the causal effects of childhood and adult BMI on C-Reactive protein and smoking behaviour.

## Author summary

Mendelian Randomisation (MR) can be used to estimate whether a potential exposure has a causal effect on an outcome in the presence of a third (unobserved) variable that affects both of them and so biases the observed association between them. The effect estimates obtained from MR studies can be interpreted as the effect of the exposure on the outcome over the lifetime. However, for some exposures there may be periods during which the causal effect on an outcome is greater or lesser than other periods. Multivariable MR (MVMR) is an extension of MR that allows for estimation of the causal effect of multiple, potentially highly related, exposures. In this paper we investigate how MVMR can be used to estimate the causal effect of the same exposure at different points across the lifecourse. We show that these effects can be estimated through MVMR when the association between the genetic variants used as instruments and the exposure varies between measurements of the exposures. However, we find that this estimation will not necessarily identify exact time periods over which an exposure has the most effect on the outcome as different periods in the lifecourse can only be separated when they are differently associated with genetic variants.

## Introduction

Mendelian Randomization (MR) uses the special properties of germline genetic variation to strengthen causal inference regarding the effect of modifiable exposures on disease. [1,2] MR can be implemented as a form of instrumental variable (IV) estimation that uses genetic variants to estimate causal effects of an exposure on an outcome that is free from bias due to unmeasured confounding. [3–5] As genetic variants which do not change during an individual’s lifetime are used as instruments the estimated effects are interpreted as the effect of the genetically predicted exposure over the lifetime, or genetic liability to an exposure if that exposure is binary. [6] Under the assumption of ‘gene-environment equivalence’, i.e. that the effect of the exposure on the outcome is the same whether variation in the exposure is due to genetic or environmental variation, the effect estimates obtained by MR can be interpreted as the effect of variation in the exposure on the outcome. [2,7]

Many exposures, such as BMI, may have varying effects on any particular outcome over the course of an individual’s lifetime. [8] Higher BMI in childhood is observationally associated with many health outcomes later in life. However whether this is due to a direct causal effect of childhood BMI on those outcomes or the high correlation between childhood and adult BMI with the latter having a causal effect on the outcome, is unclear. [9–12] If a time-varying exposure only affects a (time-invariant) outcome during a particular period then intervening on the exposure during other periods will not have any effect on the outcome. Conventional observational studies often use a lifecourse approach with the intention of identifying the particular period(s) in life that affect an outcome. [13] For example, observational studies have shown that sunlight (and from this inferred vitamin D level) in childhood, but not adulthood, is associated with risk of multiple sclerosis. [14–16] Therefore, in order to prevent multiple sclerosis it could be important to focus on time spent outside during childhood; intervening in this way during adulthood would not have any effect on the incidence of multiple sclerosis. A lifecourse approach contrasts with a MR approach which will generally provide evidence of a causal effect of the exposure on the outcome regardless of when in the lifecourse the exposure is measured. [17] For example, MR studies have shown a causal effect of vitamin D levels in the aetiology of multiple sclerosis [18] but have not identified which period is important.

When the association between genetic variants and an exposure vary over different points in the lifecourse MR estimates can be interpreted as the genetically predicted liability underlying the entire exposure history up to the time the outcome occurs. [19] That is; the effect estimated will be the effect on the outcome of having a genetic liability for the exposure that results in a one unit higher level of the exposure at the time the exposure is measured. If the genetic variants have a (proportionally) constant effect on the exposure across the entire lifetime this will be the genetically predicted lifetime effect of having a unit higher level of the exposure across the lifecourse. [20]

In this paper we explore the use of multivariable Mendelian randomization (MVMR) to estimate the causal effect of a single exposure measured at different time points in an individual’s lifetime on an outcome measured at a single fixed point in time. Structural mean models have previously been proposed for estimation of MR models with a time varying exposure. [21,22] The interpretation of the results from estimation of structural mean models will depend on the availability of data for the time-varying exposure, particularly how many time points data are available for. [21] MVMR can be implemented when multiple measures of the exposures at different points in the lifecourse are available and can be used to estimate direct effects of the exposure at each of those time points, conditional on the other time points included in the estimation. The effects estimated by MR and MVMR are described in Box 1. We outline a model for MR with an exposure measured at multiple time points and explain how this can be estimated with MVMR. We consider specific examples where the assumptions of this model do not hold and present simulation results to investigate what happens in these settings. From these results we explain how the results of such a MVMR estimation can be interpreted. We illustrate these results with application to estimating the effect of child and adulthood BMI on circulating C-reactive protein (CRP) and smoking behaviour. The results presented here show that it is possible to estimate genetically predicted direct causal effects of different time periods of an exposure on an outcome using MVMR, however careful interpretation of any results obtained from such an analysis is required. Prior knowledge regarding the biological basis of exposure trajectories can help interpretation.

**Box 1 –Effects estimated by MVMR with a time-varying exposure**

MVMR is an extension of MR that can be used to estimate the causal effects of multiple, potentially highly related, exposures. [23] The causal effect estimated by MR for an exposure that can only occur once (e.g. birthweight or age at menarche) is the total effect of the exposure on the outcome. The causal effects estimated by MVMR are the direct effect of each exposure that is not mediated by any of the other exposures included in the estimation. [24]

MR with one measure of a time-varying exposure estimates the total lifetime effect of liability to the exposure on the outcome. [19] This will include any effect on the outcome that acts through the exposure at other time periods. As we illustrate here, MVMR with multiple measures of a time-varying exposure estimates the direct effect of the liability to exposure at a particular period, i.e. the effect of the liability to the exposure at a time point that is not mediated by other time points included in the estimation. MVMR with a time-varying exposure can be implemented in the same way as MVMR with different exposures, as outlined in the methods section and in more detail elsewhere. [23,25]

## Methods

### Ethics statement

UK biobank has received ethical approval from the UK National Health Service’s National Research Ethics Service (re 11/NW/0382). All other data analysed were from publicly available summary statistics generated using relevant ethical approval from their respective studies.

**Fig 1:**
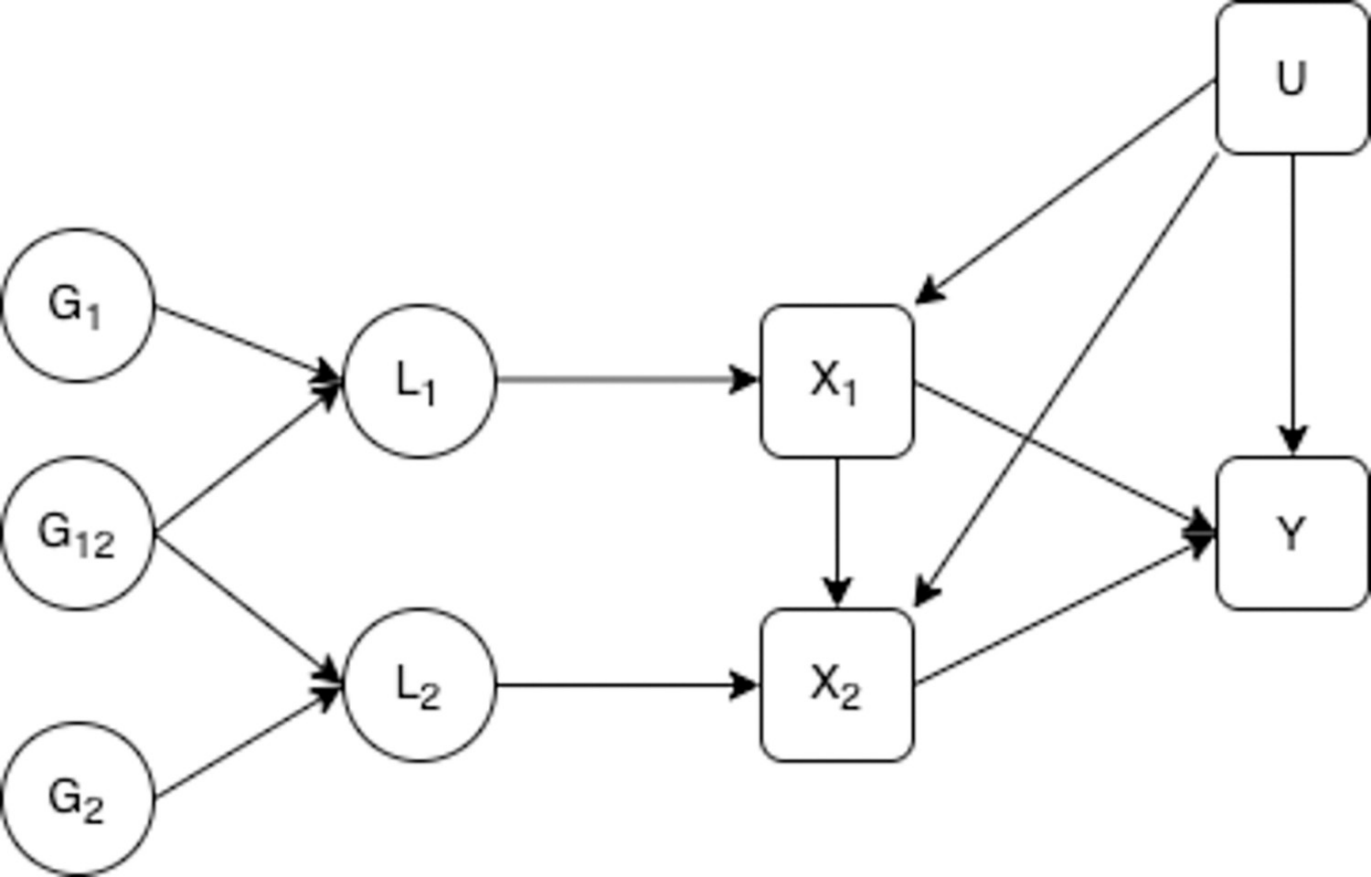
Liability exposure model with two periods of exposure. *L*_1_ is the earlier liability, *L*_2_ is the later liability, *G*_1_ is a set of genetic variants associated with *L*_1_, *G*_2_ is a set of genetic variants associated with *L*_2_, *G*_12_ is a set of genetic variants associated with both *L*_1_ and *L*_2_. *X*_1_ is a measure of the exposure in the early time period *X*_2_ is a measure of the exposure at the second time period, *Y* is an outcome observed at one time only, *U* is a set of unobserved confounders of the exposure at each time period and the outcome. *X*_1_ and *X*_2_ are potentially measured with error, error in this measurement is uncorrelated with the genetic variants.

We consider a model where genetic variants are associated with an unmeasured genetic liability for the exposure of interest which is associated with the observed value of the exposure. In this context we use the term genetic liability for the exposure to refer to the collective effect of all genetic variants associated with the exposure. [19] This liability may differ in different periods of an individual’s lifetime, however the observed trait is likely to change on a more frequent basis reflecting more short term variation and measurement error. Traits that vary across the lifecourse may have multiple liabilities that act on the exposure in different ways at different points in the lifecourse. Each liability is determined by the genetic effects and earlier liability levels do not have effects on later liability levels, although they maybe correlated through shared genetic influences and unobserved confounding. The exposure is influenced by the liability as well as confounders and other environmental influences and as Fig 1 suggests, also the earlier levels of the exposure. These exposures have a direct effect on the outcome of interest as well an effect on the subsequent value of the exposure in following periods. The genetic variants used as IVs are associated with liability in at least one period of the lifecourse but do not have to have the same association with liability in different periods. This allows for the association between a genetic variant and an exposure to vary across ages. This model is given in Fig 1. IV estimation can correct for the bias introduced by measurement error in the exposure under the assumption that the instrument is not associated with the level of that measurement error. [26] We therefore assume that the exposures are measured without error. All of the variables included (other than the individual genetic variants) are assumed to be continuous. For simplicity we initially limit the number of liability periods to two, however this model could be generalised to any number of periods. We have also excluded an effect of unobserved confounding on the liability. This simplification makes no difference to the results obtained.

### Estimation with MVMR

MVMR can be used to estimate the effect of liability to the exposure during each period, given the liability to the exposure at all of the other time points included in the estimation, i.e. the effect of *L*_1_ and *L*_2_ in Fig 1. MVMR can be conducted with either individual level data or summary data and so it is possible to use the methods described here with either type of data. Estimation using individual level data requires a dataset with the exposure measured at all time points considered and the outcome. Summary data from another non-overlapping dataset is required to enable selection of SNPs for use as instruments, conventionally those which are genome-wide significant in a GWAS study. Estimation using summary data requires SNP-exposure effects and SNP-outcome effects taken from separate samples. SNP-exposure associations for the different time points can be taken from either the same or different datasets. Analysis using summary level data is more likely to be feasible in many cases, given the large datasets required and multiple observations at different time points, so we focus here on summary level analysis.

MVMR can be implemented in a summary data setting using estimates of the association between each SNP and: the outcome, $\left(\hat{\Gamma }\right)$; exposure at one time point *X*_1_, ${(\hat{\pi }}_{1})$; and the exposure at another time point *X*_2_, $\left({\hat{\pi }}_{2}\right)$, by fitting the following model:

$$\hat{\Gamma }=\beta_{1}{\hat{\pi }}_{1}+\beta_{2}{\hat{\pi }}_{2}+\epsilon$$

Weighted by $1/{{\hat{\sigma }}_{\Gamma }}^{2}$, the inverse variance of $\hat{\Gamma }$. This approach to MVMR is a straightforward generalization of the IVW estimation framework for MR. [23,25]

MVMR estimation relies on three assumptions for estimating the causal effect of liability to the exposure at each point on the outcome. [23] These assumptions mirror the standard assumptions required for IV estimation and are that; 1. liability to each exposure is robustly predicted by the genetic variants conditional on the other exposures included in the estimation, 2. there is no confounding of the genetic variants and the outcome and 3. the genetic variants are not associated with the outcome other than via liabilities to exposures included in the estimation, i.e. there are no horizontal pleiotropic effects of the genetic variants on the outcome via other phenotypes. We address the potential for the genetic variants to affect the outcome through liability at time points not included in the estimation in our simulation results.

The first MVMR assumption implies that the exposure at each time period included in the estimation is associated with a different liability, although those liabilities may be correlated. This assumption can be tested with a conditional F-statistic. [27,28] As well as having an F-statistic at each time point greater than 10 to indicate that the genetic variants are strongly associated with that exposure, it is necessary for the conditional F-statistics to be greater than 10, indicating that the genetic variants are robustly associated with liability to exposure at each time period conditional on their association with liability to exposure at the other time periods included in the estimation.

A heterogeneity Q-statistic can be used to test for violations of the third IV assumption in the MVMR estimation. [23,27] One potential reason for excessive heterogeneity is that some of the SNPs may be associated with the outcome through pathways that are not included in the MVMR estimation, i.e. there is horizontal pleiotropy. This pleiotropy will bias the results obtained from inverse variance weighted MVMR estimation. [29,30] If pleiotropy is suspected, alternative estimation methods can be used to estimate MVMR causal effects under different assumptions of the form the pleiotropy takes. [27,30,31]

All IV estimation requires additional assumptions for interpretation of the point estimates obtained as causal effects. Firstly, all the MR and MVMR methods implemented here assume that the causal effects of the exposure(s) on the outcome are linear and, for MVMR, that there are no interactions between the effects of the exposures. Secondly, a ‘point-identifying’ assumption is required. Common point identifying assumptions for univariable IV estimation include homogeneity and monotonicity. [2] The exact definition of this point identifying assumption will determine the precise causal effect estimated, however, it is not currently well-understood how these assumptions relate to estimation with multiple exposures as in MVMR.

### Verification and comparison

#### Inclusion of exposures associated with different liability periods

**Fig 2:**
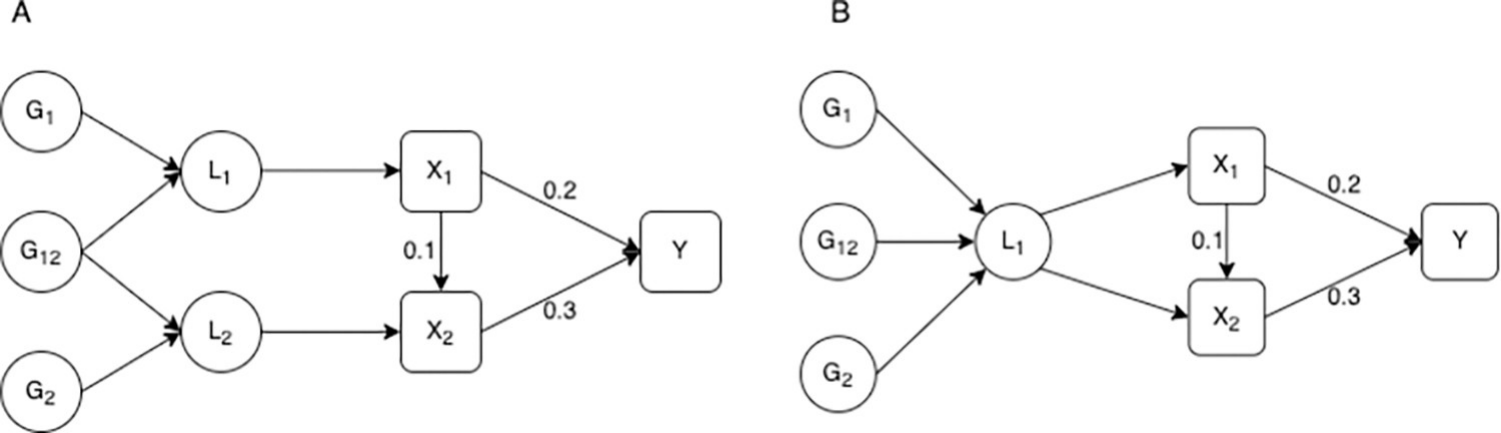
Models with different relationships between the genetic variants and the exposure at each time point. **–** *L*_1_ is the liability in the first time period, *L*_2_ is the liability in the second period. *G*_1_ is a set of genetic variants associated with *L*_1_, *G*_2_ is a set of genetic variants associated with *L*_2_ and *G*_12_ is a set of genetic variants associated with both *L*_1_ and *L*_2_. *X*_1_ and *X*_2_ are observed values of the exposure, where *X*_2_ is observed at a later point in an individual’s life than *X*_1_. *Y* is an outcome. *X*_1_, *X*_2_ and *Y* are confounded by a set of unobserved confounders *U*. In (a) *X*_1_ and *X*_2_ are associated with different liabilities. In (b) *X*_1_ and *X*_2_ are associated with the same liability. *X*_1_ and *X*_2_ are measured with error, though this measurement error is uncorrelated with the genetic variants. The direct causal effect of *X*_1_ on *X*_2_ and *Y*, and *X*_2_ on *Y* are given on the digram.

We illustrate the requirement for genetic separation in the included time periods with a simulation. We have included an exposure measured at two time points, where both measures of the exposure have a direct causal effect on the outcome and the exposure at the earlier time point also has a small direct effect on the exposure at the later time point. Following the liability model described in Fig 1 we consider two different structures for the relationship between the genetic variants, the liability and the observed value of the exposure. In the first setting each observed exposure is associated with a different underlying liability and the genetic variants have different (but correlated) effects on each liability. In the second setting both the observed exposures are associated with the same liability. This means that the genetic variants have the same effect on the exposure at each time point. These models are illustrated in Fig 2.

In each simulation we included 150 SNPs, two measures of the continuous exposures and a single continuous outcome. Unobserved confounding was modelled as two continuous variables that affected the earlier exposure measurement and the outcome or the later exposure measurement and the outcome and were excluded from the estimation. These confounders were highly correlated (rho = 0.8). The data simulated were used to generate summary associations between the SNPs and each exposure from the same sample and for the outcome using a second sample, drawn from the same population. The true association between the SNPs and each liability was normally distributed around 0 with variance 0.1/*l* where *l* is the number of SNPs. Effects of the SNPs on each liability were correlated with *ρ* = 0.25. SNPs associated with the exposure of interest for the MR estimation, or either exposure for the MVMR, with p-values < 5×10^−8^ in the exposure sample where included in the estimation. Effect estimates were obtained through inverse variance weighting MVMR (IVW–MVMR). [25] The simulations had a sample size of 150,000 and 2000 repetitions.

Results for the model with either one or two underlying liabilities are given in Table 1. These results show that the univariable estimates give an estimate of the total effect of a liability that is associated with having a unit higher level of the exposure at the time point associated with the measured exposure. This is larger than either the direct or total effect of the exposure at either time point on the outcome (given in Fig 2), due to the correlation between the genetic effects on the exposure at each time period. For example, for the first simulation given in Table 1 the direct effect of *X*_1_ on *Y* is 0.20, the total effect is 0.23 and the genetically predicted total effect is 0.34, due to an additional effect of the genetic variants on *X*_2_ which then has an effect on *Y*.

**Table 1:** Simulation results under different relationships between the genetic variants and the exposure at each time point.

		MR	MVMR
*Exposures associated with different liability periods*			
***β***_**1**_	***Liability effect***	**0.344**	**0.200**
	*Effect estimate*	0.340	0.1958
	*Est*. *Std*. *Error*	0.029	0.0107
	*Simulation Std*. *Error*	0.011	0.0106
	*Absolute bias*	0.010	0.0092
	*Coverage*	100%	93%
	*F-statistic*	96.31	
	*Conditional F-statistic*		55.76
	*No*. *SNPs*	72	114
***β***_**2**_	***Liability effect***	**0.376**	**0.300**
	*Effect estimate*	0.371	0.297
	*Est*. *Std*. *Error*	0.015	0.009
	*Simulation Std*. *Error*	0.008	0.009
	*Absolute bias*	0.008	0.008
	*Coverage*	99%	94%
	*F-statistic*	129.31	
	*Conditional F-statistic*		78.01
	*No*. *SNPs*	83	114
***Exposures associated with the same liability period***			
***β***_**1**_	***Liability effect***	**0.530**	**0.200**
	*Effect estimate*	0.519	0.207
	*Est*. *Std*. *Error*	0.011	0.080
	*Simulation Std*. *Error*	0.011	0.080
	*Absolute bias*	0.013	0.063
	*Coverage*	82%	94%
	*F-statistic*	96.31	
	*Conditional F-statistic*		1.06
	*No*. *SNPs*	72	86
***β***_**2**_	***Liability effect***	**0.480**	**0.300**
	*Effect estimate*	0.474	0.288
	*Est*. *Std*. *Error*	0.009	0.073
	*Simulation Std*. *Error*	0.009	0.072
	*Absolute bias*	0.009	0.058
	*Coverage*	89%	94%
	*F-statistic*	115.76	
	*Conditional F-statistic*		1.06
	*No*. *SNPs*	83	86

N = 150,000, reps = 2000, Direct effect of exposures on the outcome; *X*_1_: *β*_1_ = 0.2, *X*_2_: *β*_2_ = 0.3. Effect of X_1_ on X_2_ = 0.1. Effect of the liability for each exposure point are given in the table. Absolute bias is the mean value of the absolute bias of the effect estimate across the simulations. For each of *Effect estimate*, *Est*. *Std Error*, *F-statistic* and *Conditional F-statistic* mean values across each iteration of the simulation are reported. *Simulation Std*. *Error* is the estimated standard error in the effect estimate across the repetitions in the simulation. *Coverage* gives the proportion of times the true effect estimate falls within the 95%CI, *No*. *SNPs* is the mean number of SNPS selected for estimation.

When the measured exposures are associated with different liabilities, MVMR consistently estimates the genetically predicted causal effect of being on a trajectory associated with a unit higher level of that exposure, given the liability to the exposure at the other time period. However, when the measured exposures are associated with the same liability there is no difference in the genetic effects on the measured exposures and therefore weak instrument bias is introduced into the MVMR estimation. [27] This is highlighted through low conditional F-statistics. In this setting there is random variation in the direction of the bias for each exposure in each repetition of the simulation. Therefore, the mean point estimate is close to the true value of the causal effect. However, the high mean level of absolute bias shows the bias from conditionally weak instruments. This highlights how the MVMR estimates are not only biased by weak instruments but that the bias could act in either direction, with different repetitions within the same simulation being biased in opposite directions. Coverage in these simulations remains high due to the large standard error in the estimation resulting from the weak instruments, however this imprecision means total uncertainty in the simulations is high.

We additionally explored the effect of only selecting genetic variants which had differing effects at each time point on the results obtained for each of the models described here, as has previously been applied elsewhere. [32] This analysis shows that although this causes apparent conditional instrument strength to increase the causal effect estimates are potentially biased due to genetic variants which differ in the effects on each exposure more than others by chance by being selected for the analysis. These estimation results have lower power than those using all SNPs due to the reduction in the number of genetic variants included. We therefore recommend that this approach is avoided and do not consider it further. Results from this estimation and a full description of the analysis are given in S1 Text.

#### Estimation in the presence of a causal effect from the outcome to the later time point

**Fig 3:**
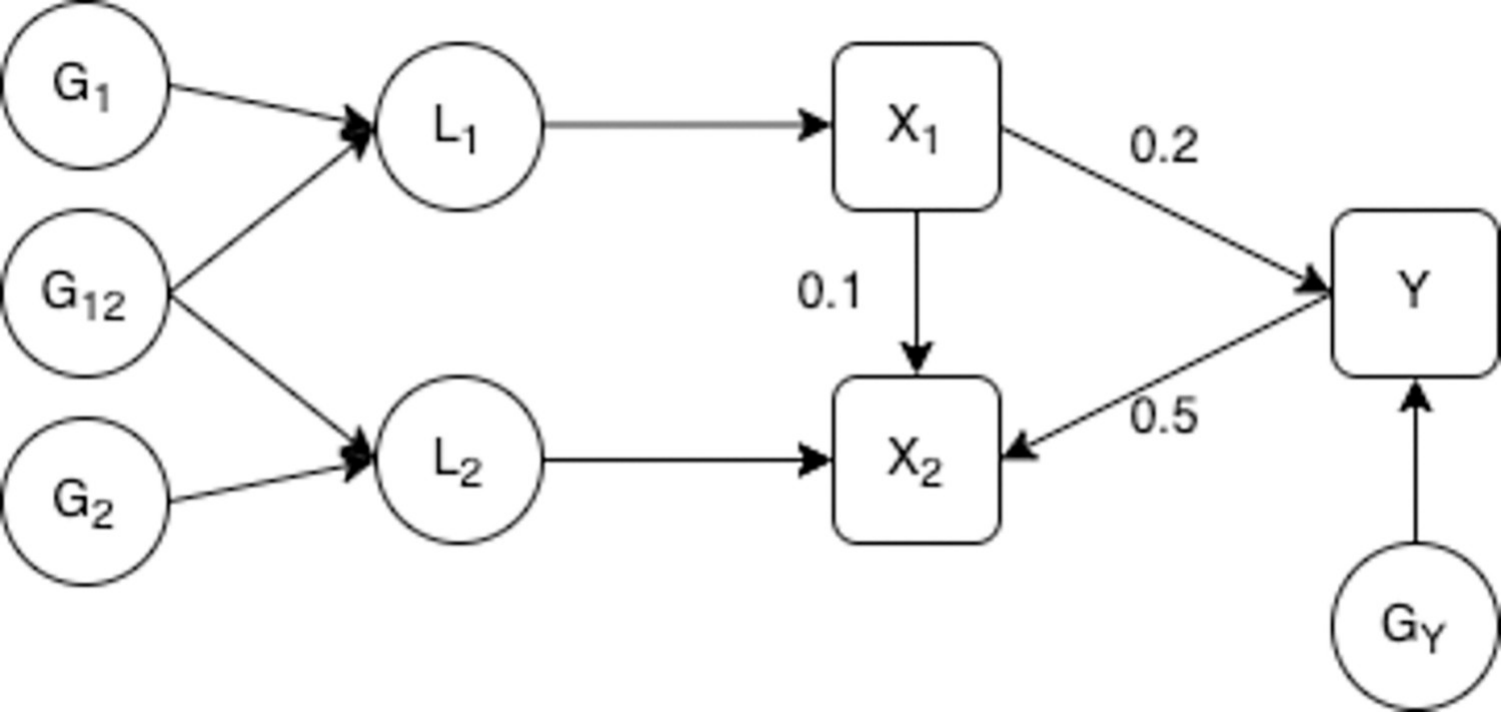
Model with a causal effect from the outcome to the later time point. **–** *L*_1_ is the liability in the first time period, *L*_2_ is the liability in the second period. *G*_1_ is a set of genetic variants associated with *L*_1_, *G*_2_ is a set of genetic variants associated with *L*_2_ and *G*_12_ is a set of genetic variants associated with both *L*_1_ and *L*_2_. *G*_*Y*_ is a set of genetic variants associated with the outcome. *X*_1_ and *X*_2_ are observed values of the exposure, where *X*_2_ is observed at a later point in an individual’s life than *X*_1_. *Y* is an outcome. *X*_1_, *X*_2_ and *Y* are confounded by a set of unobserved confounders *U*. *X*_1_ and *X*_2_ are measured with error, this measurement error is uncorrelated with the genetic variants. The direct causal effect of *X*_1_ on *X*_2_ and *Y*, and *Y* on *X*_2_ are given on the diagram.

We now consider a model where the outcome has an effect on the exposure measured at the later time point. The exposure at the later time period is therefore a collider of the earlier exposure and the outcome. This is illustrated in Fig 3 and in all other aspects the model is the same as that described in Fig 2A. Morris et al (2021) showed that estimation of this scenario with MR gives consistent estimates when there is a single underlying liability. [19] Here we consider MVMR estimation of a model with two underlying liabilities.

Simulations were set up in the same way as described for Table 1 with the addition of 50 SNPs included that were associated with the outcome *Y*. This model was estimated assuming that *X*_1_ and *X*_2_ are the true exposures and *Y* is the true outcome. All genetic variants associated with the exposure at either time period, selected based on a p-value for the SNP–exposure association of <5x10^-8^, reflecting genome-wide significance, were included in the MVMR estimation. Therefore, some SNPs strongly associated with *Y* were selected as instruments for the later time period. The model was estimated twice; firstly with no additional restrictions on the SNPs selected and secondly with Steiger filtering applied to remove any SNPs that explain more variation in the outcome than the later exposure. [33] Results from this simulation are given in Table 2.

**Table 2:** Simulation results for multiple time points with a causal effect from the outcome to the later time point.

		MR	MVMR
*No Steiger filtering*			
***β***_**1**_	***Liability effect***	**0.200**	**0.200**
	*Effect estimate*	0.196	0.078
	*Est*. *Std*. *Error*	0.016	0.070
	*Simulation Std*. *Error*	0.016	0.022
	*Absolute bias*	0.013	0.122
	*Coverage*	93%	75%
	*F-statistic*	96.34	
	*Conditional F-statistic*		59.85
	*No*. *SNPs*	72	117
***β***_**2**_	***Liability effect***	**0.076**	**0.000**
	*Effect Estimate*	0.223	0.189
	*Est*. *Std*. *Error*	0.056	0.055
	*Simulation Std*. *Error*	0.018	0.021
	*Absolute bias*	0.147	0.189
	*Coverage*	2%	0%
	*F-statistic*	101.72	
	*Conditional F-statistic*		70.82
	*No*. *SNPs*	82	117
*With Steiger filtering*			0.200
***β***_**1**_	***Liability effect***	**0.200**	**0.200**
	*Effect estimate*	0.195	0.195
	*Est*. *Std*. *Error*	0.016	0.018
	*Simulation Std*. *Error*	0.016	0.018
	*Absolute bias*	0.013	0.015
	*Coverage*	92%	94%
	*F-statistic*	96.35	
	*Conditional F-statistic*		63.68
	*No*. *SNPs*	72	107
***β***_**2**_	***Liability effect***	**0.076**	**0.000**
	*Effect Estimate*	0.083	0.001
	*Est*. *Std*. *Error*	0.017	0.015
	*Simulation Std*. *Error*	0.013	0.015
	*Absolute bias*	0.012	0.012
	*Coverage*	97%	94%
	*F-statistic*	106.80	
	*Conditional F-statistic*		69.98
	*No*. *SNPs*	72	107

N = 150,000 reps = 2000, Effect of exposure *X*_1_ on *Y*; *β*_1_ = 0.2. Effect of *Y* on *X*_2_ = 0.5. Effect of the liability for each exposure point are given in the table. Absolute bias is the mean value of the absolute bias of the genetically predicted effect estimate across the simulations. For each of *Effect estimate*, *Est*. *Std Error*, *F-statistic* and *Conditional F-statistic* mean values across each iteration of the simulation are reported. *Simulation Std*. *Error* is the estimated standard error in the effect estimate across the repetitions in the simulation. *Coverage* gives the proportion of times the true effect estimate falls within the 95%CI, *No*. *SNPs* is the mean number of SNPS selected for estimation.

Simulation results without Steiger filtering show that although the genetic variants strongly predict the exposure at each time period conditional on the other, MVMR estimation gives biased estimates of the direct causal effect of the exposure at both time periods on *Y*. This bias is due to conditioning on a variable that depends on both the exposure and the outcome (a collider) in the estimation, introducing collider bias. [34–37] Because the genetically predicted value of *X*_2_ depends on genetic variants associated with *Y*, *X*_2_ becomes the collider in the MVMR estimation. Conditioning on a collider distorts the estimated association between the other exposure and the outcome and so means that the estimates obtained in the MVMR are no longer reliable estimates of the direct effect of the earlier exposure on the outcome. Importantly, the introduction of collider bias in this estimation biases the effect estimates at each time point included in the estimation, including the earlier time point which is not dependent on *Y*.

Sanderson et al. (2019) showed that MVMR conditioning on a collider does not introduce collider bias when only genetic variants associated with the exposures are included in the estimation. [23] The different result here occurs because we have allowed for genetic variants associated with Y to be included as instruments, which was not the case in Sanderson et al. (2019) and reflects a situation where the primary phenotype has been mis-specified. [3] When Steiger filtering is applied to the results given in Table 2 there is no bias in the results obtained, as the genetic variants are restricted to those which affect the exposure at either time point directly without acting via the outcome.

### Additional excluded liability

**Fig 4:**
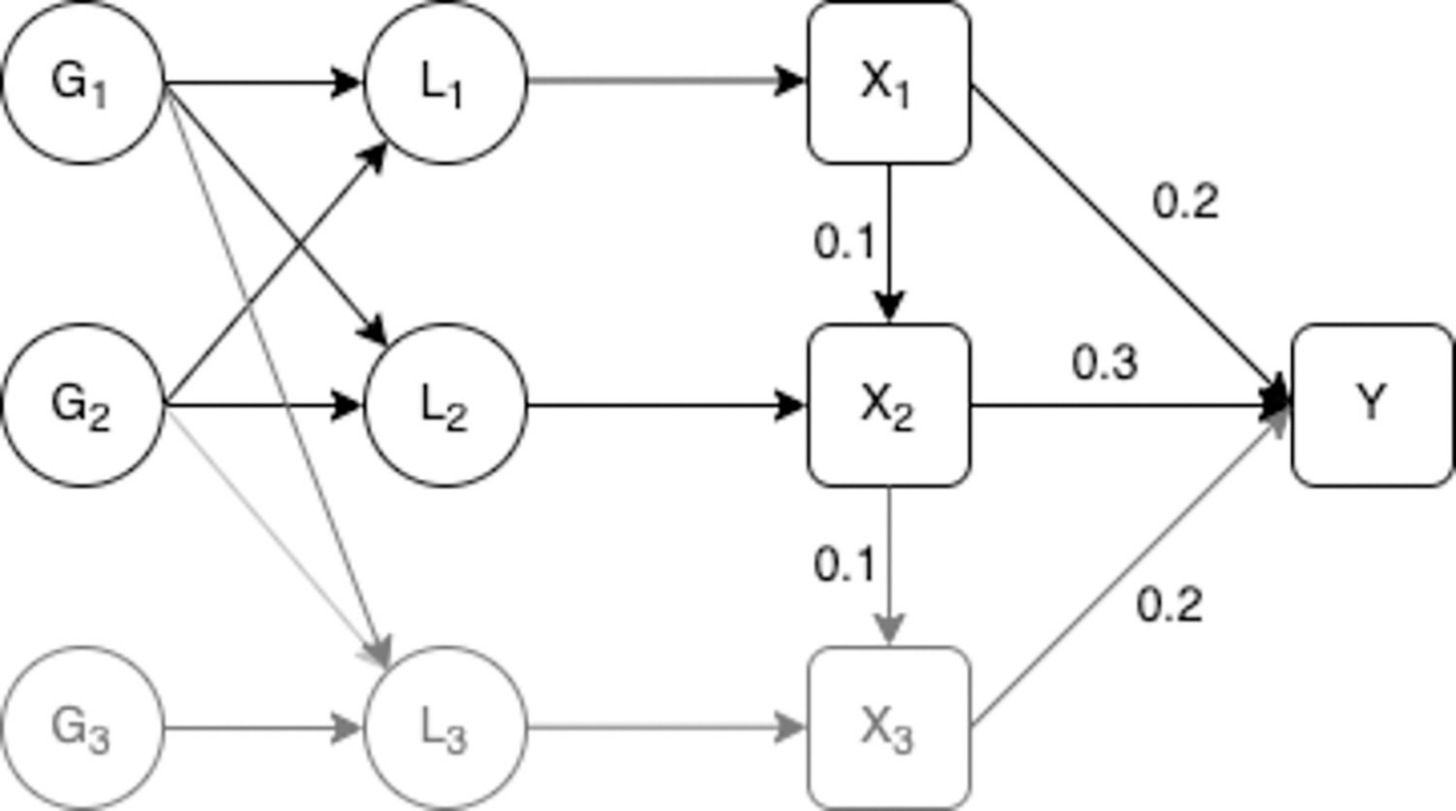
Model with three liability time periods. **–** *L*_1_ is the liability of the exposure in the first time period, *L*_2_ is the liability of the exposure in the second period, *L*_3_ is the liability of the exposure in the third period, *G*_1_ is a set of genetic variants associated with *L*_1_, *L*_2_ and *L*_3_, *G*_2_ is another set of genetic variants associated with *L*_1_, *L*_2_ and *L*_3_ and *G*_3_ is a set of genetic variants associated with *L*_3_. *X*_1_ and *X*_2_ are observed values of the exposure, where *X*_2_ is observed at a later point in an individual’s life than *X*_1_. *X*_3_ is a third value of the exposure, *X*_3_, *L*_3_ and *G*_3_ are given in grey to illustrate that although they have an effect on the outcome they are not included in the estimation. *Y* is an outcome. *X*_1_, *X*_2_, *X*_3_ and *Y* are confounded by a set of unobserved confounders *U*. *X*_1_, *X*_2_ and *X*_3_ are measured with error, this measurement error is uncorrelated with the genetic variants. The direct causal effect of *X*_1_ on *X*_2_ and *Y*, *X*_2_ on *X*_3_ and *Y*, and *X*_3_ on *Y* are given on the diagram.

We finally consider a model where the exposure has three underlying liabilities associated with it but where the model estimated only includes the exposure at times associated with two of those liabilities. The true structure of the data is illustrated in Fig 4 however the model estimated is assumed to be the same as that given in Fig 2A.

We set the simulations up in the same way as described for Table 1 with the addition of a third liability time period associated with a measured value of the exposure. This third measured exposure is assumed to be dependent on *X*_2_ and subject to overlapping confounding to both *X*_1_ and *X*_2_ with *Y*. We considered two models for the effect of *G* on *L*_3_. In the first there is no correlation between the association between *G* and *L*_3_ and the association between *G* and the other liabilities. In the second correlation between the association between *G* and *L*_3_ and *G* and *L*_1_ and *L*_2_ was added with higher correlation between *G*−*L*_2_ and *G*−*L*_3_ (*ρ* = 0.25) and a lower level of correlation between *G*−*L*_1_ and *G*−*L*_3_ (*ρ* = 0.1). These correlations arise from the overlap in the genetic effects on each liability. In both cases the outcome is assumed to occur at or after the time at which *X*_3_ is measured and all exposures have a direct causal effect on the outcome. The results from this simulation are given in Table 3.

**Table 3:** Simulation results with a relevant liability period excluded.

			MR	MVMR
*Correlated genetic effects*				
***β***_**1**_	***Liability effect***		**0.363**	**0.191**
	*Effect estimate*		0.326	0.186
	*Est*. *Std*. *Error*		0.031	0.020
	*Simulation Std*. *Error*		0.015	0.013
	*Absolute bias*		0.037	0.011
	*Coverage*		95%	100%
	*F-statistic*		88.50	
	*Conditional F-statistic*			54.82
	*No*. *SNPs*		59	93
***β***_**2**_	***Liability effect***		**0.428**	**0.353**
	*Effect estimate*		0.418	0.351
	*Est*. *Std*. *Error*		0.024	0.020
	*Simulation Std*. *Error*		0.012	0.013
	*Absolute bias*		0.013	0.010
	*Coverage*		100%	100%
	*F-statistic*		102.40	
	*Conditional F-statistic*			63.66
	*No*. *SNPs*		60	93
***Independent genetic effects***				
***β***_**1**_	***Liability effect***		**0.378**	**0.220**
	*Effect Estimate*		0.321	0.211
	*Est*. *Std*. *Error*		0.037	0.024
	*Simulation Std*. *Error*		0.015	0.014
	*Absolute bias*		0.057	0.013
	*Coverage*		80%	100%
	*F-statistic*		80.20	
	*Conditional F-statistic*			48.00
	*No*. *SNPs*		53	92
***β***_**2**_	***Liability effect***		**0.395**	**0.328**
	*Effect estimate*		0.386	0.322
	*Est*. *Std*. *Error*		0.027	0.022
	*Simulation Std*. *Error*		0.013	0.013
	*Absolute bias*		0.013	0.011
	*Coverage*		100%	100%
	*F-statistic*		98.63	
	*Conditional F-statistic*			66.32
	*No*. *SNPs*		62	92

N = 150,000 reps = 2000, *β*_1_ = 0.2, *β*_2_ = 0.3. Total effect of the exposures; *X*_1_ on *Y* = 0.232, *X*_2_ on *Y* = 0.320. Effect of the liability for each exposure are given in the table. Absolute bias is the mean value of the absolute bias of the effect estimate across the simulations. For each of *Effect estimate*, *Est*. *Std Error*, *F-statistic* and *Conditional F-statistic* mean values across each iteration of the simulation are reported. *Simulation Std*. *Error* is the estimated standard error in the effect estimate across the repetitions in the simulation. *Coverage* gives the proportion of times the true effect estimate falls within the 95%CI, *No*. *SNPs* is the mean number of SNPS selected for estimation.

When the association between the genetic variants and the excluded liability are correlated with those for the included periods the effect estimated will include some of the effect that acts via the omitted liability. The estimated effect of liability to *X*_1_ and *X*_2_ both consistently estimate of the effect of the liability at that time point. When the genetic effects on *L*_3_ are uncorrelated with the included liability periods the effect estimated does not include the effect of the later liability. Additional simulations with no direct causal effect of *X*_2_ on *Y* showed the same pattern of results and are given in Table B in S1 Text.

We finally simulated data where only *X*_2_ had a causal effect on the outcome but *X*_1_ and *X*_3_ (but not *X*_2_) were included in the estimation. In this set up we varied the association between each of *L*_1_ and *L*_3_ with *L*_2_. Results from estimation of this model showed the same pattern of results as above. The liability effect estimated includes the effect of that time period and some of the effect of the excluded time period, with the proportion of the effect of the excluded period included in the estimated effect depending on the genetic correlation between the two periods. Results from these simulations are given in Table C in S1 Text.

### Application

We consider an illustrative application where we estimate the effect of childhood and adult body mass index (BMI) on circulating C-reactive protein levels (CRP) and smoking behaviour, measured as smoking initiation, smoking cessation and cigarettes per day.

### Data

Data on child and adulthood BMI were taken from the UK biobank (UKB) study. [38,39] Between 2006 and 2010, the UK Biobank study enrolled 500,000 individuals aged between 40 and 69 at baseline across 22 assessment centres in the UK. Data were collected on clinical examinations, assays of biological samples, detailed information regarding self-reported health characteristics and genome-wide genotyping. In total 12,370,749 genetic variants in up to 463,005 individuals were available for analysis, as described previously. [40] For BMI we derived a measure of childhood body size using recall questionnaire data asking UKB participants if they were ‘thinner’, ‘plumper’ or ‘about average’ when they were aged 10 years old compared to the average. Adult body size was derived using clinically measured BMI data (mean age 56.5 years), which we categorized into a 3-category variable using the same proportion as the early life measure for comparative purposes. Genetic variants robustly associated with childhood and adult body size (based on P<5x10^-8^ and r^2^<0.001 using a reference panel from the 1000 genomes project phase 3 [41]) were identified from a previously undertaken Genome Wide Association Study (GWAS) in UKB. This GWAS has been described in-detail elsewhere as well as validation studies of the resulting genetic instruments. [8,42,43]

GWAS summary statistics for CRP levels for 204,402 European adults were extracted from Lighart et al (2018) to avoid sample overlap with UK Biobank. [44] For each of the smoking behaviour outcomes GWAS data was extracted from Lui et al (2019) using summary statistics produced excluding UKB. [45] The mean age of smoking initiation across individuals with available data (excluding UK Biobank) was 17.5 years, with the mean for each study included in the GWAS ranging from 16.0 to 21.0 years. SNPs associated with smoking initiation were identified in a sample including smokers and non-smokers. SNPs associated with smoking cessation and cigarettes per day were identified in a sample of smokers only.

For each outcome considered we estimated the genetically predicted total effect of early life and later life exposure separately through a two-sample MR using the SNPs associated with the exposure at the relevant time period. We then estimated the genetically predicted direct effects of the exposure at each time point through a MVMR estimation including both early and later life body size in the same estimation, including all SNPs associated with the exposure at either time.

## Results

Results for the estimation of the effect of BMI on CRP are given in Table 4. Our MR estimates showed a strong total effect of liability to body size in childhood and adulthood on CRP (total effect of a category increase in childhood body size on CRP (log mg/L) = 0.35, 95% CI = 0.27 to 0.42; for adult body size 0.56, 95% CI = 0.50 to 0.62) However, in the MVMR estimation no effect of early life body size liability was observed and the effect of later life liability remained largely unchanged implying that the total and direct effects of later life body size liability are similar (direct liability effect of a category increase childhood body size on circulating CRP = -0.04, 95% CI = -0.14 to 0.06, for adult body size; 0.56, 95% CI = 0.47 to 0.65).

**Table 4:** Univariable and multivariable estimates for effect of child and adulthood BMI liability on circulating CRP.

			MR–total effect			MVMR–direct effect		
	nSNPs		*β*	95% C.I.	P-value	*β*	95% C.I.	P-value
*CRP*								
age_10	190		0.35	[0.27 0.42]	2.87E-20	-0.04	[-0.14 0.06]	0.488
adult	339		0.56	[0.50 0.62]	2.62E-74	0.56	[0.47 0.65]	3.90E-38

nSNPs; number of SNPs associated with the exposure, *β*; MR effect estimate, 95% CI; 95% Confidence Interval for MR estimate, P-value; P-value for MR estimate.

Similar results were obtained for smoking behaviour, given in Table 5. MR estimates showed a strong total effect of body size liability in childhood and adulthood on all of the smoking outcomes (total effect of a category increase childhood body size on number of cigarettes per day = 0.13, 95% CI = 0.07 to 0.18, P = 2.11x10^-6^, for adult body size: 0.25, 95% CI = 0.20 to 0.30, P = 3.54x10^-26^). However, in the MVMR estimation no effect of early life body size liability on number of cigarettes per day was observed and the effect of later life liability remained largely unchanged implying that the total and direct effects of later life body size liability are similar (direct effect of a category increase childhood body size on number of cigarettes per day = -0.05, 95% CI = -0.11 to 0.01, P = 0.174, for adult body size; 0.27, 95% CI = 0.22 to 0.35, P = 7.15x10^-20^). Similar results were observed for the other smoking behaviour measures with positive total effects of liability to a higher category of childhood body size on smoking initiation and cessation observed in the MR estimation and no direct effect of childhood body size liability observed in the MVMR estimation.

**Table 5:** Univariable and multivariable estimates for effect of child and adulthood BMI liability on smoking behaviour.

			MR–total effect			MVMR–direct effect		
Exposure	nSNPs		OR	95% C.I.	P-value	OR	95% C.I.	P-value
*Smoking Initiation*								
age_10	265		1.22	[1.12 1.32]	2.35E-06	0.97	[0.86 1.09]	0.614
adult	467		1.36	[1.26 1.47]	1.77E-16	1.40	[1.27 1.55]	3.62E-11
*Smoking Cessation*								
age_10	267		1.12	[1.02 1.24]	7.18E-03	0.95	[0.83 1.09]	0.420
adult	469		1.25	[1.15 1.35]	2.69E-07	1.31	[1.16 1.47]	2.83E-06
	**nSNPs**		***β***	**95% C.I.**	**P-value**	***β***	**95% C.I.**	**P-value**
*Cigarettes per day*								
age_10	266		0.12	[0.06 0.18]	4.71E-06	-0.05	[-0.11 0.01]	0.174
adult	467		0.24	[0.20 0.27]	7.05E-28	0.27	[0.21 0.33]	7.15E-20

nSNPs; number of SNPs associated with the exposure, OR; MR estimated odds ratio for binary outcomes, *β*; MR effect estimate for continuous outcome, 95% CI; 95% Confidence Interval for MR estimate, P-value; P-value for MR estimate.

These results suggest that there is no direct effect of childhood body size liability on CRP or smoking behaviour in later life conditional on later life body size. The observed effect in the MR estimates of childhood body size on CRP and smoking are due to a combination of the effect of SNPs associated with childhood body size also having an effect on adult body size and an indirect effect of childhood body size on CRP and smoking behaviour through its effect on adult body size. Steiger filtering [33] between adult body size and the outcome removed 10 SNPs for CRP and ≤5 SNPs for any of the smoking behaviours and did not change the results obtained, results given in S1 Text.

We have not explored the potential for biases that often arise in MR and MVMR studies in the results presented here, such as biases due to pleiotropy or selection bias. [29,36,46] SNPs for smoking cessation and cigarettes per day were identified in smokers only. This leads to the potential for collider bias in the GWAS results, which would then bias the MR and MVMR results given here. These results should therefore be taken as an illustration of the application and interpretation of the methods discussed.

## Discussion

When multiple measures of an exposure at different time points are available, MVMR can be used to estimate the causal effect of changing the liability of the exposure at different time points on the outcome. The interpretation of the MVMR estimate is the direct effect of having a liability associated with a unit higher level of the exposure at that time point, for a given liability for the exposure at the other time points included in the estimation. That is, the effect of having a liability associated with a unit higher level of *X*_1_ while keeping the liability for *X*_2_ constant. If measures of the exposure at different time periods are available, it is possible to identify whether the exposure effects persist over time or key periods exist in the lifecourse.

As shown in simulation results given in Table 1, an important restriction for estimation of these models is that the association between the genetic variants and the exposure must vary over the periods included in the estimation. Although genetic variants themselves do not vary over an individual’s lifetime, variation in their effects could arise from different genetic variants having different levels of importance in the development of the exposure at different ages. In the simulations we have assumed that each liability only directly affects the exposure at one time period but that genetic variants can be associated with multiple liabilities. However, the results obtained would be the same if we had allowed each genetic variant to influence one liability only but for the liabilities to affect the exposure at multiple time periods and each exposure to be influenced by multiple liabilities.

Our simulation results highlight how it is possible to introduce collider bias to the results obtained when genetic variants for the outcome are selected as instruments for the exposure. Steiger filtering should be applied to help remove this bias if there is potential for the outcome to mediate some of the relationship between the exposure at the time periods included in the estimation.

Our final set of simulations show how the effects of any time periods excluded from the estimation but associated with genetic variants included in the estimation will form part of the effect estimated. The size of this effect will depend on the level of correlation between the liabilities for the included and excluded time periods. It is likely that for many exposures the genetic variants associated with the trait at one time point will also be associated with the trait at another time point to some degree. Therefore, an observed effect for one time period may not be due to the exact time period measured. If MVMR is being used to identify which periods in the lifecourse are most important, then the other potentially important periods also need to be included in the estimation.

**Fig 5:**
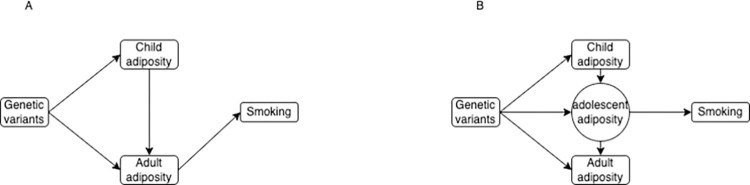
Estimated relationship between adiposity and smoking behaviour. **–** Figure shows (a) the estimated relationship between adiposity and smoking behaviour and (b) a potential underlying model that would give this result.

Our application to smoking behaviour illustrates how genetic correlation between time points can mean that a particular point can appear important even though any effect is likely to have occurred earlier in the lifecourse. The results obtained show an effect of adult body size liability (mean age: 56.5) on smoking initiation (mean age: 17.5 years) once childhood body size liability has been controlled for. Typical age of smoking initiation therefore precedes the measurement of adulthood body size. The large effect of body size liability at the age measured in our sample on risk of smoking is unlikely to be causal at the point of time that the exposure and outcome were measured. If liability for higher body size in adulthood is associated with liability for higher body size in adolescence/early adulthood the effect we estimate may reflect the effect of body size in adolescence on smoking initiation even though it is actually measured at a later time point. This model is illustrated in Fig 5. Data on BMI at different ages between childhood and adulthood would potentially enable estimation of the effect on smoking behaviour at a range of different ages and so identification the period between childhood and adulthood that was most important in the development of smoking behaviour. Implementation of this approach with MVMR would however rely on those periods being differentially associated with the genetic variants used as instruments. This difference has been shown previously for body mass index (BMI) at different points across childhood and between childhood and adulthood. [8,47,48] However other research has shown that the genetic influences on BMI are consistent across adulthood. [49] This would prevent using this approach to determine which point in adulthood is most important for risk of smoking behaviour.

Previous work using this estimation approach has shown that early life BMI liability does not have a direct effect on type 2 diabetes and coronary heart disease. [8] Therefore if an individual with a high BMI in early life reduces their excess weight in later life their risk for type 2 diabetes and coronary heart disease will not be increased via this pathway. Our analysis of the effect of body size on circulating CRP levels show a similar result, larger body size in early life will not have an effect on increased CRP levels if the excess weight is reduced in adulthood. These results however do not identify the periods in adulthood that are most important.

The methods described here also require the general assumptions of MR estimation to hold. The assumption that all of the data is from the same underlying population is important to all summary-data MR analysis. [50,51] This is likely to be particularly important when considering the same exposure at different ages as changes in the distribution of the exposure or the relationship between the exposure and the outcome between different cohorts could potentially bias the results obtained. The choice of datasets should be carefully considered if the same data cannot be used for each time point. For example; the distribution of childhood BMI levels has changed notably over the last 50 years and therefore it would not be correct to assume that BMI measured in groups of adults and children at the same point in time would represent measures from the same population.

If some SNPs are differently associated with more than one time period for the exposure and the causal effect of the exposure on the outcome varies over time there may be heterogeneity in the results obtained, even in the absence of pleiotropy. In this case those SNPs that have a larger association with the exposure in the time period with the largest causal effect will estimate a larger causal effect of the exposure on the outcome. This will inflate the heterogeneity Q-statistic even in the absence of conventional pleiotropic effects, unless all relevant time periods are included in the estimation.

Throughout this work we have only considered a single measurement of the outcome. For many exposures and outcomes it may be possible that the outcome could also vary over time with the relationship between the exposure and outcome varying at different time points, and potentially also effects of earlier values of the exposure on later values of the outcome. This type of relationship, with multiple different outcomes, cannot be estimated with standard MVMR methods. This is therefore left as an area of future research.

## Supporting information

S1 Text Supplementary material. Table B–No direct causal effect of X_2._ Table C–Additional simulation results for a model with three exposure periods.
(DOCX)
