# Supplementary material for: Estimation of causal effects of a time-varying exposure at multiple time points through multivariable mendelian randomization
Source: PLoS Genet. 2022 Jul 18;18(7):e1010290. doi: 10.1371/journal.pgen.1010290 (PMC9348730; doi:10.1371/journal.pgen.1010290)
Supplement: S1 Text — Table B–No direct causal effect of X2. Table C–Additional simulation results for a model with three exposure periods. (DOCX) [file pgen.1010290.s001.docx]

**Estimation of causal effects of a time-varying exposure at multiple time points through Multivariable Mendelian randomization.**

**SUPPLEMENTARY MATERIAL**

Eleanor Sanderson^1,2^, Tom G Richardson^1,2,3^, Tim T Morris^1,2^, Kate Tilling^1,2^, George Davey Smith^1,2^

1. *MRC Integrative Epidemiology Unit at the University of Bristol.*
2. *Population Health Sciences, Bristol Medical School, University of Bristol.*
3. *Novo Nordisk Research Centre, Headington, Oxford, OX3 7FZ, United Kingdom*

**Section 1. Selected variants used for estimation**

Table A repeats the estimation results given in Table 1 with an additional preselection applied to the genetic variants for the MVMR estimation. In this estimation genetic variants have been selected that have the largest difference in effect on each time period only. These were defined as those in the top 20% for the absolute difference of effect on the exposure in each period.

**Table A –** *Simulation results under different relationships between the genetic variants and the exposure at each time point with selected variants.*

|  |  | **MR** | **MVMR** |
| --- | --- | --- | --- |
| ***Exposures associated with different latent periods*** | |  |  |
| $\boldsymbol{\beta}_{\boldsymbol{1}}$ | ***Genetically predicted lifetime effect*** | **0.344** | **0.200** |
|  | *Effect estimate* | 0.340 | 0.196 |
|  | *Est. Std. Error* | 0.029 | 0.021 |
|  | *Simulation Std. Error* | 0.011 | 0.020 |
|  | *Absolute bias* | 0.010 | 0.016 |
|  | *Coverage* | 100% | 93% |
|  | *F-statistic* | 96.31 |  |
|  | *Conditional F-statistic* |  | 62.88 |
|  | *No. SNPs* | 72 | 22 |
| $\boldsymbol{\beta}_{\boldsymbol{2}}$ | ***Genetically predicted lifetime effect*** | **0.376** | **0.300** |
|  | *Effect estimate* | 0.371 | 0.297 |
|  | *Est. Std. Error* | 0.015 | 0.016 |
|  | *Simulation Std. Error* | 0.008 | 0.016 |
|  | *Absolute bias* | 0.008 | 0.013 |
|  | *Coverage* | 99% | 95% |
|  | *F-statistic* | 129.31 |  |
|  | *Conditional F-statistic* |  | 94.60 |
|  |  | 83 | 22 |
| ***Exposures associated with the same latent period*** | |  |  |
| $\boldsymbol{\beta}_{\boldsymbol{1}}$ | ***Genetically predicted lifetime effect*** | **0.530** | **0.200** |
|  | *Effect estimate* | 0.519 | 0.200 |
|  | *Est. Std. Error* | 0.011 | 0.170 |
|  | *Simulation Std. Error* | 0.011 | 0.190 |
|  | *Absolute bias* | 0.013 | 0.140 |
|  | *Coverage* | 82% | 94% |
|  | *F-statistic* | 96.31 |  |
|  | *Conditional F-statistic* |  | 1.40 |
|  |  | 72 | 18 |
| $\boldsymbol{\beta}_{\boldsymbol{2}}$ | ***Genetically predicted lifetime effect*** | **0.480** | **0.300** |
|  | *Effect estimate* | 0.474 | 0.300 |
|  | *Est. Std. Error* | 0.009 | 0.140 |
|  | *Simulation Std. Error* | 0.009 | 0.150 |
|  | *Absolute bias* | 0.009 | 0.120 |
|  | *Coverage* | 89% | 94% |
|  | *F-statistic* | 115.76 |  |
|  | *Conditional F-statistic* |  | 1.41 |
|  |  | 83 | 18 |

N= 150,000, reps = 2000, $\beta_{1}=0.2$, $\beta_{2}=0.3$. Effect of X_1_ on X_2_ = 0.1. True genetically predicted effects for each estimation are given in the table. Absolute bias is the mean value of the absolute bias of the effect estimate across the simulations. For each of *Effect estimate, Est. Std Error, F-statistic* and *Conditional F-statistic* mean values across each iteration of the simulation are reported. *Simulation Std. Error* is the estimated standard error in the effect estimate across the repetitions in the simulation.

**Section 2. Additional simulation results for a model with three exposure periods**

***No causal effect of X_2_***

We replicated the simulations given in Table 2 with the direct causal effect of X_2_ set to zero. Otherwise, the model is the same as given in Table 2 in the main paper.

These results show the same pattern of results as given in the main paper. Both MR and MVMR estimate the lifetime effect of the exposure, however this will differ from the direct causal effect at a particular time point when the genetic variants used as instruments are associated with a period excluded from the estimation.

**Table B – No direct causal effect of X_2_**

|  |  | **MR** | **MVMR** |
| --- | --- | --- | --- |
| $\boldsymbol{\beta}_{\boldsymbol{1}}$ | ***Genetically predicted lifetime effect*** | **0.315** | **0.291** |
|  | *Effect estimate* | 0.309 | 0.283 |
|  | *Est. Std. Error* | 0.018 | 0.019 |
|  | *Simulation Std. Error* | 0.011 | 0.011 |
|  | *Absolute bias* | 0.010 | 0.011 |
|  | *Coverage* | 100% | 100% |
|  | *F-statistic* | 88.50 |  |
|  | *Conditional F-statistic* |  | 54.83 |
|  | *No. SNPs* | 59 | 93 |
| $\boldsymbol{\beta}_{\boldsymbol{2}}$ | ***Genetically predicted lifetime effect*** | **0.166** | **0.053** |
|  | *Effect estimate* | 0.160 | 0.057 |
|  | *Est. Std. Error* | 0.029 | 0.019 |
|  | *Simulation Std. Error* | 0.011 | 0.011 |
|  | *Absolute bias* | 0.010 | 0.009 |
|  | *Coverage* | 100% | 100% |
|  | *F-statistic* | 102.40 |  |
|  | *Conditional F-statistic* |  | 63.66 |
|  | *No. SNPs* | 60 | 93 |

N= 150,000, reps = 1000, $\beta_{1}=0$, $\beta_{2}=0.3$, $\beta_{3}=0$. Effect of X_1_ on X_2_ = 0.1, effect of X_2_ on X_3_ = 0.1. True genetically predicted effects for each estimation are given in the table. Absolute bias is the mean value of the absolute bias of the effect estimate across the simulations. For each of *Effect estimate, Est. Std Error, F-statistic* and *Conditional F-statistic* mean values across each iteration of the simulation are reported. *Simulation Std. Error* is the estimated standard error in the effect estimate across the repetitions in the simulation. *Coverage* gives the proportion of times the true effect estimate falls within the 95%CI, *No. SNPs* is the mean number of SNPS selected for estimation.

***Only a causal effect of X2***

The model is set up with the causal effects given Figure A, otherwise the model estimated is the same as given in Table 3 in the main paper. However, the model is estimated excluding X_2_, so only X_1_ and X_3_ (which do not have a direct causal effect) are included in the estimation. Three different scenarios were considered for this model; a) similar genetic correlations between the second period and each of the first and third periods, b) stronger genetic correlation between the first and second periods and weaker genetic correlation between the second and third periods and c) weaker genetic correlation between the first and second periods and stronger genetic correlation between the second and third periods.

The results given in this table show that the model estimates the direct causal effect of each time period, including a share of the effect that acts through the excluded second period. When the genetic correlations are varied the period which has larger correlation with the excluded period has the largest causal effect.

**Figure A**

**

| **Scenario a** | $\boldsymbol{\rho}_{\boldsymbol{12}}$**=0.3** | $\boldsymbol{\rho}_{\boldsymbol{23}}$**=0.3** |
| --- | --- | --- |
| **Scenario b** | $\boldsymbol{\rho}_{\boldsymbol{12}}$**=0.5** | $\boldsymbol{\rho}_{\boldsymbol{23}}$**=0.2** |
| **Scenario c** | $\boldsymbol{\rho}_{\boldsymbol{12}}$**=0.2** | $\boldsymbol{\rho}_{\boldsymbol{23}}$**=0.5** |

$\boldsymbol{\rho}_{\boldsymbol{12}}$ **– genetic correlation between X_1_ and X_2_**

$\boldsymbol{\rho}_{\boldsymbol{23}}$ **– genetic correlation between X_2_ and X_3_**

**Table C –** *Additional simulation results for a model with three exposure periods.*

1. *Similar correlations between each period*

|  |  | **MR** | **MVMR** |
| --- | --- | --- | --- |
| $\boldsymbol{\beta}_{\boldsymbol{1}}$ | ***Genetically predicted lifetime effect*** | **0.137** | **0.108** |
|  | *Effect estimate* | 0.115 | 0.099 |
|  | *Est. Std. Error* | 0.027 | 0.027 |
|  | *Simulation Std. Error* | 0.016 | 0.015 |
|  | *Absolute bias* | 0.023 | 0.014 |
|  | *Coverage* | 97% | 100% |
|  | *F-statistic* | 88.53 |  |
|  | *Conditional F-statistic* |  | 61.11 |
|  | *No. SNPs* | 59 | 93 |
| $\boldsymbol{\beta}_{\boldsymbol{3}}$ | ***Genetically predicted lifetime effect*** | **0.117** | **0.116** |
|  | *Effect estimate* | 0.119 | 0.105 |
|  | *Est. Std. Error* | 0.032 | 0.030 |
|  | *Simulation Std. Error* | 0.018 | 0.017 |
|  | *Absolute bias* | 0.014 | 0.016 |
|  | *Coverage* | 100% | 100% |
|  | *F-statistic* | 86.77 |  |
|  | *Conditional F-statistic* |  | 55.70 |
|  | *No. SNPs* | 54 | 93 |

1. *Stronger genetic correlation between X_1_ and X_2_*

|  |  | **MR** | **MVMR** |
| --- | --- | --- | --- |
| $\boldsymbol{\beta}_{\boldsymbol{1}}$ | ***Genetically predicted lifetime effect*** | 0.197 | 0.172 |
|  | *Effect estimate* | 0.166 | 0.165 |
|  | *Est. Std. Error* | 0.027 | 0.027 |
|  | *Simulation Std. Error* | 0.016 | 0.016 |
|  | *Absolute bias* | 0.031 | 0.014 |
|  | *Coverage* | 90% | 100% |
|  | *F-statistic* | 83.556 |  |
|  | *Conditional F-statistic* |  | 55.642 |
|  | *No. SNPs* | 57.034 | 92.291 |
| $\boldsymbol{\beta}_{\boldsymbol{3}}$ | ***Genetically predicted lifetime effect*** | 0.076 | 0.092 |
|  | *Effect estimate* | 0.089 | 0.074 |
|  | *Est. Std. Error* | 0.032 | 0.028 |
|  | *Simulation Std. Error* | 0.017 | 0.016 |
|  | *Absolute bias* | 0.017 | 0.020 |
|  | *Coverage* | 100% | 98% |
|  | *F-statistic* | 90.84 |  |
|  | *Conditional F-statistic* |  | 59.34 |
|  | *No. SNPs* | 55.976 | 92.291 |

1. *Stronger genetic correlation between X_2_ and X_3_*

|  |  | **MR** | **MVMR** |
| --- | --- | --- | --- |
| $\boldsymbol{\beta}_{\boldsymbol{1}}$ | ***Genetically predicted lifetime effect*** | **0.104** | **0.096** |
|  | *Effect estimate* | 0.082 | 0.076 |
|  | *Est. Std. Error* | 0.035 | 0.028 |
|  | *Simulation Std. Error* | 0.019 | 0.017 |
|  | *Absolute bias* | 0.024 | 0.022 |
|  | *Coverage* | 100% | 98% |
|  | *F-statistic* | 80.16 |  |
|  | *Conditional F-statistic* |  | 49.25 |
|  | *No. SNPs* | 54 | 94 |
| $\boldsymbol{\beta}_{\boldsymbol{3}}$ | ***Genetically predicted lifetime effect*** | **0.155** | **0.162** |
|  | *Effect estimate* | 0.149 | 0.156 |
|  | *Est. Std. Error* | 0.024 | 0.025 |
|  | *Simulation Std. Error* | 0.015 | 0.015 |
|  | *Absolute bias* | 0.013 | 0.013 |
|  | *Coverage* | 100% | 99% |
|  | *F-statistic* | 94.24 |  |
|  | *Conditional F-statistic* |  | 66.78 |
|  | *No. SNPs* | 64 | 94 |

N= 150,000, reps = 1000, $\beta_{1}=0$, $\beta_{2}=0.3$, $\beta_{3}=0$. Effect of X_1_ on X_2_ = 0.1, effect of X_2_ on X_3_ = 0.1. True genetically predicted effects for each estimation are given in the table. Absolute bias is the mean value of the absolute bias of the effect estimate across the simulations. For each of *Effect estimate, Est. Std Error, F-statistic* and *Conditional F-statistic* mean values across each iteration of the simulation are reported. *Simulation Std. Error* is the estimated standard error in the effect estimate across the repetitions in the simulation. *Coverage* gives the proportion of times the true effect estimate falls within the 95%CI, *No. SNPs* is the mean number of SNPS selected for estimation.

**Section 3. Application results with steiger filtering applied**

**Table D –** *Univariable and multivariable estimates for effect of child and adulthood BMI on circulating CRP with Steiger filtering applied*

*Steiger filtering has been applied to remove any SNPs that predict more variation in adult BMI category than in circulating CRP, 10 SNPs were removed due to this.*

|  | |  | **MR – total effect** | | | **MVMR – direct effect** | | |
| --- | --- | --- | --- | --- | --- | --- | --- | --- |
|  | **nSNPs** | | $\boldsymbol{\beta}$ | **95% C.I.** | **P-value** | $\boldsymbol{\beta}$ | **95% C.I.** | **P-value** |
| *CRP* | | | |  |  |  |  |  |
| age_10 | 190 | | 0.35 | [0.28 0.42] | 2.87E-20 | -0.03 | [-0.12 0.05] | 0.444 |
| adult | 329 | | 0.54 | [0.48 0.58] | 1.92E-92 | 0.55 | [0.47 0.63] | 1.11E-43 |

nSNPs; number of SNPs associated with the exposure, $\beta$; MR effect estimate, 95% CI; 95% Confidence Interval for MR estimate, P-value; P-value for MR estimate.

**Table E –** *Univariable and multivariable estimates for effect of child and adulthood BMI on smoking behaviour with Steiger filtering applied.*

*Steiger filtering has been applied to remove any SNPs that predict more variation in adult BMI category than in the smoking outcome considered. No SNPs were removed for smoking initiation, 1 SNP was removed for smoking cessation and 5 SNPs for Cigarettes per day.*

|  | | **MR – total effect** | | | | | **MVMR – direct effect** | | | | |
| --- | --- | --- | --- | --- | --- | --- | --- | --- | --- | --- | --- |
| **Exposure** | **nSNPs** | **OR** | | **95% C.I.** | | **P-value** | **OR** | | **95% C.I.** | **P-value** | |
| *Smoking Initiation* | |  | |  | |  |  | |  |  | |
| age_10 | 265 | 1.22 | | [1.13 1.32] | | 2.35E-06 | 0.97 | | [0.86 1.09] | 0.614 | |
| adult | 467 | 1.36 | | [1.26 1.47] | | 1.77E-16 | 1.40 | | [1.27 1.55] | 3.62E-11 | |
| *Smoking Cessation* | |  | |  | |  |  | |  |  | |
| age_10 | 267 | 1.12 | | [1.02 1.24] | | 0.007 | 0.95 | | [0.85 1.07] | 0.447 | |
| adult | 468 | 1.23 | | [1.14 1.33] | | 4.76E-07 | 1.30 | | [1.15 1.46] | 4.89E-06 | |
|  | **nSNPs** | | $\boldsymbol{\beta}$ | **Std. Err** | **P-value** | | | $\boldsymbol{\beta}$ | **Std. Err** | | **P-value** |
| *Cigarettes per day* | | |  |  |  | | |  |  | |  |
| age_10 | 265 | | 0.11 | [0.05 0.17] | 6.80E-06 | | | -0.04 | [-0.10 0.02] | | 0.252 |
| adult | 462 | | 0.23 | [0.19 0.27] | 6.76E-27 | | | 0.26 | [0.20 0.32] | | 1.34E-18 |

nSNPs; number of SNPs associated with the exposure, OR; MR estimated odds ratio for binary outcomes, $\beta$; MR effect estimate for continuous outcome, 95% CI; 95% Confidence Interval for MR estimate, P-value; P-value for MR estimate.
